# A Quasi‐Solid‐State Tristate Reversible Electrochemical Mirror Device with Enhanced Stability

**DOI:** 10.1002/advs.201903198

**Published:** 2020-05-12

**Authors:** Alice Lee‐Sie Eh, Jingwei Chen, Shu Hearn Yu, Gurunathan Thangavel, Xinran Zhou, Guofa Cai, Shaohui Li, Daniel H. C. Chua, Pooi See Lee

**Affiliations:** ^1^ School of Materials Science and Engineering Nanyang Technological University 50 Nanyang Avenue Singapore 639798 Singapore; ^2^ Singapore‐HUJ Alliance for Research and Enterprise (SHARE) Nanomaterials for Energy and Energy Water Nexus (NEW) Campus for Research Excellence and Technological Enterprise (CREATE) Singapore 138602 Singapore; ^3^ Material Sciences and Engineering Department National University of Singapore Singapore 117575 Singapore

**Keywords:** cycling stability, electrochemical mediators, electrochromic devices, reversible electrochemical mirrors, reversible electrodeposition

## Abstract

Reversible electrochemical mirror (REM) electrochromic devices with electrochemical tunability in multiple optical states are exciting alternatives to conventional electrochromic smart windows. Electrochromic devices are studied extensively, yet widespread adoptions have not been achieved due to problems associated with durability, switching speed, limited options on optical states, and cost. In this study, a REM electrochromic device based on CuSn alloy is developed, which offers highly reversible switching between transparent, greyish‐blue, and mirror states via reversible electrodeposition and dissolution. The alloying element, Sn acts as an electrochemical mediator, which facilitates the electrodeposition and dissolution of Cu. The CuSn‐based REM device shows superior cycling stability for 2400 cycles (transmittance mode) and 1000 cycles (reflectance mode). The electrodeposited CuSn alloy film is resistant to surface oxidation in ambient air, with a 2.9% difference in reflectance at 2000 nm after 3 days. In addition, the alloy film exhibits excellent NIR reflectance property with thermal modulation of 18.5 °C at a high temperature of 180 °C. The REM device with zero power consumption maintains its mirror state for at least 100 min, making it a promising candidate for energy‐efficient applications.

## Graphical abstract

The quasi‐solid‐state tristate reversible electrochemical mirror (REM) device offers highly reversible switching between transparent, greyish‐blue, and mirror states via reversible electrodeposition and dissolution. The REM shows superior thermal barrier property compared to the bare substrate. The extended memory effect of the REM device is achieved in the presence of quasi‐solid‐state polymer electrolyte.

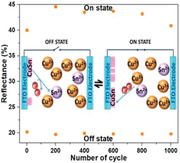

## 1 Introduction

Electrochromics, which enable dynamic control of solar heat and lighting, have found widespread successful commercialization as smart glass technology that has been installed in architectural buildings, automobiles,^[^ ^1^ ^]^ and aircraft.^[^ ^2^ ^]^ This aesthetic glazing technology increases the energy efficiency of buildings as it offers low energy consumption and a good coloration memory effect. Electrochromic device is able to manage incoming solar irradiation into buildings that resulted in lighting, heating, ventilation, and air‐conditioning energy savings, thereby reducing the energy consumption in the buildings when installed as smart windows.^[^ ^3^ ^]^

For the past few decades, electrochromic research has focused primarily on transition metal oxides, polymers, and small organic molecules, which can reversibly modulate their optical properties with electrical bias.^[^ ^4^ ^]^ Alternative technologies such as the up‐and‐coming switchable plasmonic metasurfaces are interesting as the color‐tunable metasurfaces offer full color gamut, high chromaticity, high spatial resolution, thus suitable for ultrathin and flexible reflective displays.^[^ ^5^ ^]^ With the current approaches, it is challenging to simultaneously achieve various optical states, high optical contrast, fast switching speed, and long‐term durability, thus delaying the widespread adoption of this sustainable technology. In recent works, researchers have explored reversible metal electrodeposition in reversible electrochemical mirrors (REMs) as promising alternatives to conventional electrochromic materials for smart windows.^[^ ^6^ ^]^ The most attractive feature of the REMs is the ability to demonstrate multiple states that include the neutral transparent state, colored state, and mirror state.^[^ ^6a–e^ ^]^ In addition, REMs offer prolonged cycling (transmittance mode) without significant degradation and fast switching speed.^[^ ^6a,c,h^ ^]^ These tunable mirrors are earmarked as highly attractive candidates for privacy glass, visor controls, electronic displays, thermal control, and camouflage.

Despite these promising features, the main challenge hindering the progress of REMs is the difficulty in achieving high cycling stability in the reflectance mode. Most of the progress in the REMs are limited to the cycling stability in the transmittance mode^[^ ^6a,d,h^ ^]^ and none has reached reflectance cycling stability beyond 50 cycles.^[^ ^6^, ^8^ ^]^ This difficulty is due to the fact that metal ions encounter partial irreversibility in the electrodeposition and dissolution steps. A number of literatures have reported the use of electrochemical mediators to help in the dissolution of metal films.^[^ ^6a,f^ ^]^

In this work, we introduce an alloying strategy to enhance the reversibility of the electrochemical deposition and dissolution in REM. Typically, silver(Ag) or copper (Cu) is used as the reversible electrodeposition metal in view of its high electroactivity, recyclability, and ubiquitous availability.^[^ ^6a,e^ ^]^ The best reported cycling reversibility of Cu REM was limited to 200 cycles in transmittance mode, as found in our prior work.^[^ ^6e^ ^]^ In our current approach, the selected alloying element should possess higher reduction potential (more positive potential), with respect to the parent metal, so that it gets electrodeposited first to form the nucleation layer, followed by electrodeposition of the parent metal. The alloying element should also possess lower oxidation potential than the parent host metal in order to initiate dissolution of the alloy film, followed by complete film dissolution during the bleaching state in order to enhance the reversibility of the REM.

Herein, tin (Sn) is judiciously selected as the alloying element as it meets the requirements of having close redox potentials with Cu (standard electrode potential difference of ≈0.48 V), exhibiting high reflectance and is stable in air.^[^ ^7^ ^]^ We achieved a tristate quasi‐solid‐state CuSn‐based REM device with enhanced cycling stability, attaining 2400 cycles between the transparent and colored mode and 1000 cycles in the reflectance mode, surpassing the current state‐of‐the‐art.^[^ ^8^ ^]^ The presence of Sn as the alloying element is vital in achieving robust reversibility as it facilitates the electrodeposition and dissolution of Cu mirror film. Additionally, the presence of Sn endows resistance against the surface oxidation of Cu at the ambient environment, leading to higher reflectivity of the CuSn alloy film (compared to Cu) when subjected to extended air exposure. Extended memory effect of the REM device has been achieved in the presence of quasi‐solid‐state of the polymer electrolyte containing poly(vinyl alcohol) (PVA), hindering the fast dissolution of the CuSn alloy. Our prototype REM device could be scaled up with ease up to 64 cm^2^ and three devices were programmed by applied voltage to simultaneously display the tristates namely, transparent, greyish‐blue, and mirror states.

## 2 Results and Discussion

### 2.1 Electrochemical Characterization

**Figure 1:**
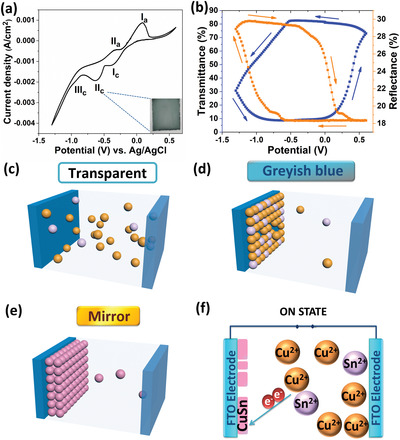
a) Cyclic voltammogram of the CuSn alloy film electrodeposition/dissolution on the FTO working electrode in the electrolyte in the potential range from −1.30 to +0.60 V versus Ag/AgCl; Inset is the photograph of the film at the potential of −0.63 V; b) corresponding changes in transmittance (λ = 550 nm, left axis in blue) and reflectance (λ = 660 nm, right axis in orange), respectively, during the CV scan; c) random distribution of the Cu^2+^ (orange spheres) and Sn^2+^ (light purple spheres) ions in the quasi‐solid‐state CuSn‐based REM device when no potential is applied; d) formation of greyish‐blue film at a potential of –0.9 V; e) formation of mirror film at −1.2 V (pink spheres denote the electrodeposition of CuSn nanoparticles forming the CuSn alloy film); and f) illustration of the electrodeposition of Cu^2+^ and Sn^2+^ ions to form the CuSn alloy film, in the REM device.

In order to investigate the electrochemical reduction and oxidation behaviors of the CuSn electrolyte, cyclic voltammetry (CV) analysis of CuSn electrodeposition/dissolution on the FTO electrode was conducted at a scan rate of 20 mV s^−1^ from −1.30 to +0.60 V (vs Ag/AgCl) in a three‐electrode configuration with platinum electrode as the counter electrode (**Figure** **1**a). The respective CVs of the Cu and Sn electrolytes were analyzed individually, as shown in Figures S1 and S2, Supporting Information. From the CV curve in Figure 1a, typical redox peaks can be ascribed to the reduction and oxidation of Cu and Sn ions, leading to electrodeposition and dissolution of Cu and Sn on the FTO electrode. The first cathodic peak, *I*_c_ ≈ −0.36 V can be ascribed to the reduction of Cu^2+^ ions to Cu^+^ ions. The second cathodic peak, *II*_c_ ≈ −0.63 V corresponds to the formation of metallic Sn when Sn^2+^ ions are reduced to Sn^0^. As the cathodic sweep reaches −0.95 V, the third cathodic peak, *III*_c_ was observed. The peak *III*_c_ can be attributed to the formation of metallic copper when Cu^+^ ions undergo electrochemical reduction to Cu^0^. As the cathodic sweep continues, the reflectivity of the alloy film increases (Figure 1b, orange line) as more Cu and Sn nanoparticles are electrodeposited onto the FTO electrode. The structural and composition of the CuSn alloy film will be elucidated later in the text. When the potential is swept from −1.30 V toward the positive potential, an anodic peak *II*_a_ appears at ≈−0.32 V. The *II*_a_ peak corresponds to the oxidation of Cu^0^ to Cu^+^ ions and Sn^0^ to Sn^2+^, leading to an increase in the transmittance of the FTO electrode as the CuSn film dissolves back into the electrolyte (Figure 1b). Since the oxidation potential of Sn^0^ to Sn^2+^ is lower than that of Cu^+^ to Cu^2+^, Sn^2+^ mediates the dissolution of Cu in the film. The anodic peak *I*_a_ ≈ +0.10 V corresponds to the oxidation of Cu^+^ to Cu^2+^, indicating complete dissolution of CuSn film from the FTO electrode back into the electrolyte. Notably, the redox peak of alloying element, Sn (Sn^0^ to Sn^2+^) is positioned at ≈−0.32 V with a lower oxidation potential than the parent metal, Cu (Cu^+^ to Cu^2+^) at ≈+0.10 V, which initiates the dissolution of the CuSn alloy film. The structural and composition characterizations of the greyish‐blue and mirror films will be elucidated later. Initially, the quasi‐solid‐state CuSn electrolyte showed poor Coulombic efficiency of 5.9% during the first cycle, reaching efficiency of 28.6% after two cycles and improving tremendously to 78.6% after ten cycles (Figure S3, Supporting Information). The calculated Coulombic efficiency for the CV of the quasi‐solid‐state CuSn electrolyte is 90.5% after 2200 cycles and 85.7% after 2500 cycles, indicating good reversibility in terms of electrodeposition and dissolution of the CuSn system.

Figure 1c shows the randomly distributed Cu^2+^ and Sn^2+^ ions within the electrolyte reservoir during the voltage‐off state (transparent state). A greyish‐blue film was obtained by applying a negative potential of −0.9 V to the working FTO electrode as seen in Figure 1d. Both Cu^2+^ and Sn^2+^ ions are electrochemically reduced to Cu^0^ and Sn^0^ to form the reflective CuSn alloy film at −1.2 V (Figure 1e). Figure 1f gives a brief illustration of the electrodeposition of Cu and Sn ions to form CuSn alloy film in the REM device. The alloy film could be dissolved back into the electrolyte by applying a positive potential to the working electrode, thereby increasing the optical transmittance of the device. The electrochemical reactions for the electrodeposition of Cu and Sn ions in the CuSn solution are as follows:

Greyish‐blue film formation:

(1) $${\text{CuCl}}_{2}+{\text{e}}^{-}\to \text{CuCl}+{\text{Cl}}^{-}$$

(2) $${\text{Sn}}^{2+}+2{\text{e}}^{-}\to \text{Sn}$$

Mirror film formation:

(3) $$\text{CuCl}+{\text{SnCl}}_{2}+3{\text{e}}^{-}\to \text{CuSn}+3{\text{Cl}}^{-}$$

The CuCl and Sn films tend to oxidize at ambient environment:

(4) $$\text{CuCl}+4{\text{O}}_{2}+{\text{Cl}}^{-}\to \text{Cu}{\left({\text{ClO}}_{4}\right)}_{2}+{\text{e}}^{-}$$

(5) $$2\text{Sn}+{\text{O}}_{2}\to 2\text{SnO}$$

The electrodeposition and dissolution mechanism of the CuSn alloy can be elucidated based on the reduction and oxidation of Cu and Sn ions in the electrolyte. The formation of greyish‐blue film can be attributed to the presence of blue CuCl film and grey SnO film. The CuCl is formed when Cu^2+^ ions (CuCl_2_) are electrochemically reduced to Cu^+^ ions, which undergoes oxidation under the ambient environment to form Cu(ClO_4_)_2_, which can be detected via X‐ray diffraction (XRD) instead of CuCl. The presence of grey film is attributed to the presence of SnO. Beyond −0.95 V, both Cu and Sn ions undergo electrochemical reduction to form the CuSn alloy film.

### 2.2 Structural and Morphological Characterizations

**Figure 2:**
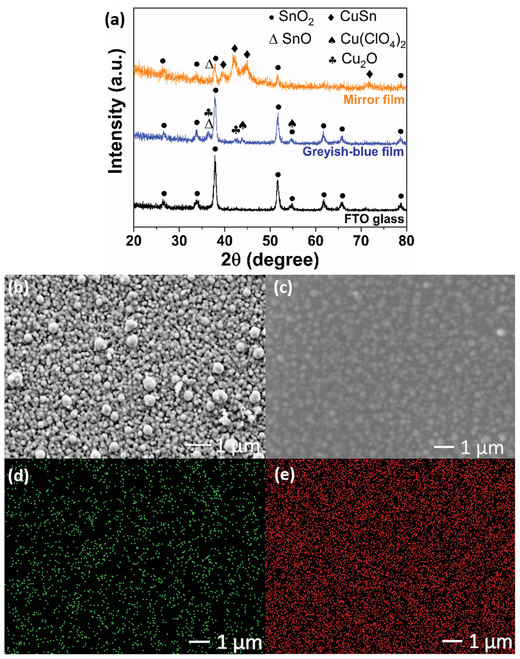
a) XRD patterns of the bare FTO electrode, electrodeposited greyish‐blue film/FTO electrode, and electrodeposited CuSn alloy mirror film/FTO electrode. b) High‐magnification FESEM images of the electrodeposited CuSn film on the FTO electrode from the CuSn electrolyte (without PVA) at −1.5 V for 60 s. c) High‐magnification FESEM images of the electrodeposited CuSn alloy film/FTO electrode from quasi‐solid‐state CuSn electrolyte (with PVA) at −1.5 V for 60 s, corresponding energy dispersive X‐ray spectroscopy mapping of d) Cu and e) Sn of the CuSn alloy film.

The greyish‐blue and mirror films were characterized by XRD. As shown in **Figure** **2**a, the XRD diffraction peaks of the greyish‐blue film can be ascribed to Cu(ClO_4_)_2_ (monoclinic, #00‐032‐0328), Cu_2_O (cubic, #00‐005‐0667), and SnO (orthorhombic, #00‐013‐0111). The presence of Cu_2_O and SnO can be attributed to the oxidation of both Cu(I) and Sn(0) in air. The XRD diffraction peaks of mirror film can be reasonably indexed to CuSn alloy (orthorhombic, #00‐006‐0621) and SnO (orthorhombic, #00‐013‐0111). Based on the relative intensity of the diffraction peaks, it is evident that CuSn is the major component in the mirror film. The morphology of the CuSn alloy film was revealed by SEM, as shown in Figure 2b. The CuSn film was electrodeposited from the electrolyte by applying a potential of −1.5 V (vs Ag/AgCl) for 60 s. The size of CuSn alloy nanoparticles ranges from 150 to 550 nm. From the EDS analysis (Figure 2c–e), the CuSn alloy film displayed homogeneous distribution of Cu and Sn elements across the film. Increasing the electrodeposition duration to 180 s resulted in the growth of larger CuSn nanoparticles up to 400–650 nm (Figure S4, Supporting Information). Ideally, electrodeposition of smaller nanoparticles size is preferred as more compact and uniform film of higher reflectivity can be obtained with minimal diffusive reflectance. To obtain a highly reflective film, one can tailor the reflectivity by optimizing the particle morphology, particle size, film thickness, uniformity of the film, surface roughness of the film, intrinsic reflectivity property of the metal, electrolyte pH, rate of electrodeposition, current density, and additives used.^[^ ^9^ ^]^ In our prior work, we have introduced PVA in the electrolyte that increased the viscosity of the electrolyte in order to slow down ion mobility and reduce the rate of electrodeposition effectively.^[^ ^6e^ ^]^ In the presence of PVA in the electrolyte, the SEM analysis showed electrodeposition at −1.5 V (vs Ag/AgCl) for 60 s resulted in smaller CuSn nanoparticles (85–150 nm) without aggregation and film thickness of about 98 nm (Figure S5, Supporting Information).

**Figure 3:**
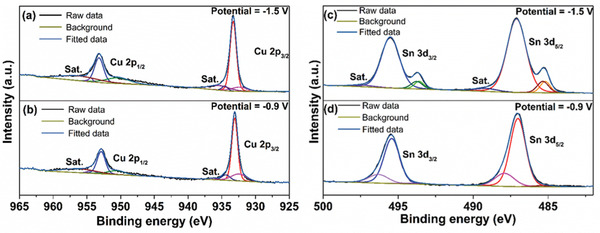
XPS spectra of a,c) Cu 2p and Sn 3d of CuSn mirror film electrodeposited at −1.5 V and b,d) greyish‐blue film electrodeposited at −0.9 V.

To understand the chemical nature of electrodeposited CuSn films, X‐ray photoelectron spectroscopy (XPS) technique was employed to analyze the samples deposited at different potentials. **Figure** **3**a,b show the deconvoluted Cu 2p_3/2_ spectra of CuSn mirror film (−1.5 V) and greyish‐blue film (−0.9 V). The partial oxidized Cu species are due to surface oxidation of pure Cu. The peak separation between Cu 2p_3/2_ and Cu 2p_1/2_ is about 19.9 eV^[^ ^10^ ^]^ with shakeup satellite Cu 2p_3/2_ peaks along 938–945 eV.^[^ ^11^ ^]^ For CuSn alloy film electrodeposited at −1.5 V (Figure 3a), the major peak located at 933.3 eV is characteristic of Cu 2p_3/2_ of Cu^0^.^[^ ^12^ ^]^ The presence of this sharp peak indicated successful electrodeposition of CuSn film of high purity. Minor peaks located at 934.5 and 932.4 eV are also deconvoluted, which are tentatively assigned to Cu 2p_3/2_ of Cu^2+^ and Cu^0^, respectively.^[^ ^13^ ^]^ As seen in Figure 3b, the major peak located at 933.1 eV is characteristic of Cu 2p_3/2_ of Cu^+^ as reported in the literature.^[^ ^14^ ^]^ This is in close agreement with our XRD result of the greyish‐blue Cu film, which revealed the presence of Cu^+^. The minor peaks located at 934.4 and 932.4 eV can be associated with Cu 2p_3/2_ of Cu^2+^ and Cu^0^.^[^ ^13^ ^]^ The presence of Cu^0^ can be explained based on the close cathodic peak of Cu (*III*_c_ ≈ −0.95 V as depicted in the CV) with the applied potential of −0.9 V for the electrodeposition of the greyish‐blue film.

Likewise, the Sn 3d spectra can also be deconvoluted into distinct peaks in order to identify the chemical environment of Sn in samples electrodeposited at various potentials. Figure 3c,d shows the high‐resolution Sn 3d XPS spectra and the representative deconvoluted Sn 3d_5/2_ spectra of CuSn mirror film (−1.5 V) and greyish‐blue film (−0.9 V). The peak separation between Sn 3d_5/2_ and Sn 3d_3/2_ is about 8.41 eV^[^ ^10^ ^]^ with shakeup satellite peaks at higher binding energy. There are two strong shoulder peaks located at 485.4 and 485.1 eV present in the Sn3d_5/2_ spectra of CuSn mirror film deposited at −1.5 V, which can be associated with Sn^0^ in Sn metal and Sn in CuSn alloy.^[^ ^12^, ^15^ ^]^ The other major peak located at 487.1 eV is tentatively assigned to Sn3d_5/2_ of Sn^2+^, due to possible oxidation in the film. At a higher electrodeposition potential of −1.5 V, Sn3d_5/2_ peak associated with Sn^4+^ was not detected due to the absence of SnO_2_ as delineated in the XRD result. The Sn3d_5/2_ spectra of greyish‐blue film can be seen in Figure 3d, the peak located at binding energy of 487.0 eV suggested the presence of Sn^2+^, in which Sn^0^ was oxidized to SnO in the presence of residual oxygen.^[^ ^16^ ^]^ At potential of −0.9 V, Sn^2+^ in the electrolyte is electrochemically reduced to Sn^0^, which explains the presence of the weak peak at 485.4 eV of Sn^0^ of Sn metal in Figure 3d.^[^ ^15^ ^]^ The peak located at 487.9 eV can be associated with Sn 3d_5/2_ of Sn^4+^ which belonged to SnO_2_.^[^ ^17^ ^]^

From the quantification of the atomic ratios obtained from the XPS analysis (Table S1, Supporting Information), the ratio of Cu:Sn is 1:1.54 for the CuSn mirror film. Sn (Sn^2+^ to Sn^0^) has a higher cathodic peak than Cu (Cu^1+^ to Cu^0^) and thus undergoes electrochemical reduction at a lower potential. When electrodeposited at a potential of −1.5 V, the decrease in the ratio of Cu:Sn is expected. For the greyish‐blue film which is electrodeposited at −0.9 V, the increase in the ratio of Cu:Sn to 1.48:1 is as expected since Cu (Cu^2+^ to Cu^1+^) has higher cathodic peak than Sn (Sn^2+^ to Sn^0^). Thus, the amount of Cu detected in the greyish‐blue film is higher compared to Sn.

### 2.3 Electrochromic Performance

Liquid electrolyte is not favorable in manufacturing process for practical, well‐sealed electrochromic devices. Foreseen issues, namely, poor chemical stability, presence of bubbles, electrolyte leakage, or hydrostatic pressure concerns are some of the solvent‐related safety issues.^[^ ^18^ ^]^ On the other hand, solid electrolyte presented mediocre electrochromic performance, poor interfacial properties, and lower ionic conductivity due to the poor mobility of the ionic species in the solid matrix.^[^ ^18^, ^19^ ^]^ Due to its non‐Newtonian nature, the quasi‐solid‐state electrolyte combines the advantages of both liquid (diffusive transport properties) and solid (cohesive properties) electrolytes, such as being easy to apply, good wettability, excellent interfacial properties, high ionic conductivity, and long‐term stability.^[^ ^18^, ^19^, ^20^ ^]^ Hence, we prepared the quasi‐solid‐state CuSn electrolyte for electrochromic performance analysis of the REM device.

**Figure 4:**
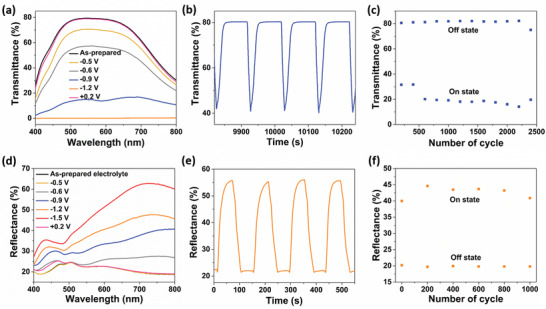
a) In situ transmittance spectra of the CuSn electrodeposited on the FTO electrode at various electrodeposition potentials in the wavelength range of 400–800 nm. b) In situ transmittance response of the electrodeposition and dissolution of the CuSn alloy film on the FTO electrode. c) Cycling performance of the quasi‐solid‐state CuSn‐based REM device for 2400 cycles using the potential algorithms (PA1) at 550 nm in the transmittance mode. d) Reflectance spectra of the CuSn electrodeposited on the FTO electrode at various electrodeposition potentials in the wavelength range of 400–800 nm. e) In situ reflectance response of the electrodeposition and dissolution of the CuSn alloy film on the FTO electrode. f) Cycling performance of the quasi‐solid‐state CuSn‐based REM device for 1000 cycles using the potential algorithms (PA2) at 660 nm in the reflectance mode.

In situ transmittance and reflectance spectroscopy analyses were conducted under various electrodeposition potentials to understand the electrochromic performance of the quasi‐solid‐state CuSn electrolyte in the REM device. The transmittance spectra of the device were measured at potentials of −0.5, −0.6, −0.9, −1.2, and +0.2 V for 60 s in the wavelength range of 400 to 800 nm, as shown in **Figure** **4**a. At its neutral state, the device showed a high transmittance of 79.33% at 550 nm, with air as the baseline. At the intermediate tinted state, a greyish‐blue film was obtained at −0.9 V, and exhibited a low transmittance of 14.8%. This tinted state is beneficial for the smart windows application as it provides indoor comfort to the building occupants while conserving the energy consumption of the buildings as very low electrical potential is required. The mirror film was obtained at −1.2 V and exhibited almost zero transmittance of 0.05%. This mirror state is suitable for privacy glass application. As shown in Figure 4a, the CuSn‐based REM device demonstrated a high transmittance modulation of 78.8% at 550 nm under the applied potentials of −1.2 and +0.2 V. From the reflectance spectra, as shown in Figure 4d, it can be seen that the mirror state is initiated at −0.6 V. At this specific potential, Sn^2+^ was electrochemically reduced to Sn^0^ to form the metallic Sn film and thus resulted in reflectance modulation in the REM device. At a higher potential of −1.5 V, the device exhibited a pronounced reflectance at wavelength of 730 nm that is, 62.79% with a reflectance modulation of 43.39% when switched between −1.5 and +0.2 V. Additionally, the absorption spectra of both greyish‐blue and mirror films were investigated (shown in Figure S6, Supporting Information, and discussed) to understand the characteristic bands related to Cu transitions and Sn charge transfer.

Switching speed is one of the most critical parameters in the electrochromic devices and determines the competitive features of the smart windows and electronic displays.^[^ ^4^, ^21^ ^]^ It explains the kinetics of the electrochemical process when transiting from one state to another under alternating potentials. Switching speed is defined as the time needed for an electrochromic system to reach 90% of its full modulation between the steady colored state and bleached state.^[^ ^22^ ^]^ The coloration and bleaching speed of the CuSn film in the quasi solid electrolyte was studied via in situ transmittance spectroscopy at the wavelength of 550 nm using the potential algorithm PA1 (−1.2 V [10 s], 0 V [60 s], +0.2 V [20 s], and 0 V [10 s]) as can be seen from Figure 3b. The potential algorithms (PA1 and PA2) are shown as waveforms in Figure S7, Supporting Information. The switching speed for coloration and bleaching was determined to be 9.8 and 18.4 s, respectively. The low transmittance indicates the formation of the CuSn film and high transmittance indicates the dissolution of the CuSn film from the FTO electrode. The cycling stability of the REM device is demonstrated in Figure 4c. Prior to cycling tests, we applied adjusted potential algorithm for activation of the freshly fabricated device in order to initiate nucleation on the bare FTO electrode. The first step is to apply higher electrodeposition potential of −1.5 V to the working FTO electrode to drive the electrodeposition of Cu and Sn, and gradually decreasing the potential to −1.2 V upon repeated tenths of cycles. The device exhibited an initial transmittance modulation of 42.5% and achieved a maximum transmittance modulation of 68.0% at 2200th cycle. The transmittance modulation of the device gradually decreases, and maintained a modulation of 55.4% after 2400 cycles (Figure 4d). Notably, the device performed better than the initial cycling, increased the transmittance modulation by 12.9% after 2400 cycles. The cycling stability result revealed the robust switching between the high transmittance and low transmittance states of the CuSn‐based REM device, which verified the excellent reversibility in the electrodeposition and dissolution of the CuSn alloy in the quasi‐solid‐state electrolyte. However, in the absence of Sn, the pure Cu‐based REM device exhibited inferior cycling stability of 400 cycles using the same potential algorithm PA1 (Figure S8a, Supporting Information). This demonstrated the significant role of tin as the alloying element in facilitating the electrodeposition and dissolution of copper.

Analysis of the REM device was conducted to investigate the factors that caused a drop in the transmittance modulation property after 2400 cycles. To understand the electrochemical reduction and oxidation behaviors of the CuSn film after the long‐time cycling, CV analysis of the CuSn electrodeposition/dissolution on the FTO electrode was carried out (Figure S9, Supporting Information). The second cathodic peak, *II*_c_ ≈ −0.71 V and third cathodic peak *III*_c_ ≈ −1.01 V shifted toward more negative potential, indicating the increased energy barrier to achieve electrochemical reduction of Sn^+^ to Sn^0^ and Cu^+^ to Cu^0^. Compared to the initial CV, both the anodic peaks, *II*_a_ ≈ −0.16 V and *I*_a_ ≈ 0.15 V shifted toward a more positive potential. The cycled device required a more negative reduction potential to achieve the mirror state and a more positive oxidation potential to achieve complete film dissolution, which explained the drop in the transmittance modulation at the 2400th cycle. After the strenuous cycling, the film morphology illustrates agglomeration of the CuSn nanoparticles, which resulted in the irregular thickness across the film (Figure S10b, Supporting Information). The nonuniform film often resulted in the drop of the transmittance modulation as the rough film is less compact, leading to diffusive state during the UV–vis–NIR spectrophotometry measurement. In addition, it will become increasingly difficult for CuSn film to undergo complete film dissolution due to the nonuniform electrical field distribution across the irregular thickness of CuSn alloy film. For the structural characterization of the CuSn alloy film, XRD analysis of the film after the strenuous cycling of 2400 cycles was conducted. The film showed no structural change and the film composition was retained (Figure S11, Supporting Information). CuSn is still the major component in the mirror film.

From Figure 4e, the switching speed for mirror film formation and dissolution was determined to be 24.7 and 30.2 s, respectively. The switching speed is considered fast for the REM device as it only took half a minute to achieve reflectance modulation of 34.8%. Figure 4f shows that the CuSn‐based REM device has an excellent cycling stability of 1000 cycles in the reflectance mode at the wavelength of 660 nm via PA2 (−1.2 V [15 s], 0 V [60 s], +0.2 V [20 s], and 0 V [15 s]). PA2 allows longer deposition and dissolution times to achieve higher film reflectivity and complete film dissolution. In the absence of Sn, the pure Cu‐based REM device showed poor cycling stability of 40 cycles (Figure S8b, Supporting Information) via the same potential algorithm PA2. In our work, we found that the presence of Sn as the alloying element is vital in achieving robust reversibility and the absence of Sn caused irreversibility in electrodeposition and dissolution of Cu mirror film, device degradation, and premature device failure.

The cycle kinetics of the quasi‐solid‐state CuSn‐based REM device at different switching cycles in the transmittance and reflectance modes were also investigated. In the transmittance mode (Figure S12a, Supporting Information), the coloration speed was slightly faster (first cycle: 8.2 s; 2400th cycle: 7.4 s) but the bleaching speed increased to 36.9 s during the 2400th cycle (first cycle: 12.7 s). In the reflectance mode (Figure S12b, Supporting Information), the time taken for mirror film formation was reduced by 2.1 s (first cycle: 13.7 s; 1000th cycle: 11.6 s) but the mirror dissolution time increased to 77.7 s during the 1000th cycle (first cycle: 15.5 s). Therefore, the main factor of the premature device degradation as observed from the cycle kinetics was due to the challenging film dissolution over time. Based on the above discussion, it is conclusive that during the cycling process, electrodeposition of CuSn alloy film remains relatively fast, which is related to undissolved CuSn nanoparticles that act as nucleation layer and facilitate the electrodeposition (shown in the Figure S13a,b, Supporting Information). The increase in the crystal nucleation frequency is also evident during the cycling test using the Johnson–Mehl–Avrami–Kolmogorov (JMAK) analysis, *X* = 1 − exp(−kt^n^) as shown in Figure S14, Supporting Information. However, the dissolution process became the rate‐limiting step, with longer bleaching/dissolution time along with the cycling process. The presence of non‐dissolved nanoparticles, in one way assists the electrodeposition process, yet in another way impedes the dissolution process and eventually leads to device degradation.

The key challenges in tailoring the ideal polymer electrolyte for REM devices are the preservation of the high ionic conductivity, high optical transparency, good chemical and electrochemical stabilities, long‐term stability, wide potential window, while retaining ease of electrolyte handling.^[^ ^4^, ^18^, ^23^ ^]^ From the practical point of view, quasi‐solid‐state electrolyte is preferred as it offers good ionic conductivity, excellent interfacial properties, ease of application, and long‐term stability.^[^ ^18^, ^20^ ^]^ The rheological behavior of the quasi‐solid‐state CuSn electrolyte was as expected for a viscoelastic fluid. The G′ (storage modulus) and G″ (loss modulus) as a function of frequency for electrolyte within linear deformation range were illustrated in Figure S15, Supporting Information. To evaluate the viscoelastic behavior of the quasi‐solid‐state electrolyte, the G′ and G″ as a function of frequencies were determined from the dynamic strain sweep tests, which demonstrated a typical elastic response of the electrolyte sample. The decrease in the dynamic viscosity of the quasi‐solid‐state electrolyte suggests that it has the rheological characteristics of shear thinning, thus allowing injection of electrolyte that facilitates ease of electrolyte handling. As shown in Figure S15, Supporting Information, the storage modulus (G′) of quasi‐solid‐state electrolyte was higher than their loss modulus (G″) under frequency sweeps, determining the slime‐like behavior (Figure S16, Supporting Information), displaying similar behaviors as reported in the literatures.^[^ ^18^, ^24^ ^]^

In general, higher ionic conductivity facilitates faster rate of electrodeposition and dissolution as ions diffusion occurs readily. The ionic conductivity of the quasi‐solid‐state CuSn electrolyte was investigated via electrochemical impedance spectroscopy (EIS), which was conducted in the frequency range from 100 kHz to 0.1 Hz at an open‐circuit potential as seen in Figure S17a, Supporting Information. Figure S17, Supporting Information, shows the Nyquist plot of the quasi‐solid‐state CuSn electrolyte with a semicircle in the high‐frequency region. The semi‐circle is ascribed to the charge transfer impedance. The EIS pattern can be fitted by the equivalent circuit with resistive and capacitive combination as shown in the inset of Figure S17a, Supporting Information. The equivalent circuit elements were deduced by fitting the experimental data with the equivalent circuit by using the ZView software. R1 represents the resistance attributed to the electrolyte and the electrodes, R2 represents the charge‐transfer resistance, and CPE2 (constant phase element) is related to the associated capacitance. Figure S17a, Supporting Information, shows the experimental data fitted well with the values calculated from the equivalent circuit. In the EIS spectrum, the quasi‐solid polymer electrolyte has an ionic conductivity of 8.46 × 10^−5^ S cm^−1^. Up‐to‐date, there is unavailable report on the ionic conductivity of the electrolyte used in the REM devices from other research groups but there are extensive studies on this important parameter for electrochromic applications. The conductivity of an electrolyte is a function of the mobility of the individual ions, degree of dissociation, electrolyte composition, viscosity, and temperature.^[^ ^25^ ^]^ From the ionic conductivity and switching speed results, it was concluded that a higher ionic conductivity promotes ion mobility, which inherently increases the rate of electrodeposition and dissolution of the CuSn alloy. Coloration efficiency (CE) is one of the most important criteria often used to characterize an electrochromic material. For conventional electrochromic devices, higher CE is preferred as less charge or energy is required to reach larger optical modulations. Similarly, for REM system, reflectance efficiency (RE) is defined as the change in optical density (∆*OD*) per unit of charge (∆*Q*) for electrodeposition or dissolution of the mirror film, where *R*_m_ and *R*_t_ denote the reflectance in the mirror and transparent states, respectively, at a fixed wavelength. It can be derived from the following formulas:

(6) $$RE\;\;\left(\lambda \right)=\;\frac{\Delta OD\;\left(\lambda \right)}{\Delta Q}$$

(7) $$\Delta OD\;\;\left(\lambda \right)=\log\frac{R_{\text{m}}}{R_{\text{t}}}\;$$

Till date, the efficiency of the REM device has not been reported. As can be seen from Figure S18, Supporting Information, the quasi‐solid‐state CuSn‐based REM device showed a reflectivity efficiency of 9.7 cm^2^ C^−1^. For REM device, it would be desirable to achieve higher reflectivity efficiency in order to increase the energy efficiency of the device for adoption of wider device applications that require low power consumption. Memory effect refers to zero current consumption of the electrochromic system after coloration. This memory effect of electrochromism is often cited as a valuable, desirable property of the electrochromic systems, which enables energy savings in green buildings. The quasi‐solid‐state CuSn‐based REM device has a good memory effect retention. In the reflectance mode, the quasi‐solid‐state CuSn‐based REM device exhibited a significant memory effect of 100 min (at 660 nm) upon the application of −1.5 V for 3 min as shown in Figure S19a, Supporting Information. Park et al. demonstrated a long memory effect of 120 min in a silver‐based REM device by applying longer electrodeposition time of 30 min at higher potential of −2.5 V.^[^ ^6g^ ^]^ The memory effect of our REM device was attributed to the quasi‐solid‐state of the polymer electrolyte that acted as an effective barrier in slowing down the diffusion of anions into CuSn film and prevented the CuSn film from dissolving back into the electrolyte during the voltage‐off state. In the greyish‐blue state, the device demonstrated appreciable optical memory of 14.5 min (at 550 nm) upon the application of −0.9 V for 3 min as can be seen from Figure S19b, Supporting Information.

**Figure 5:**
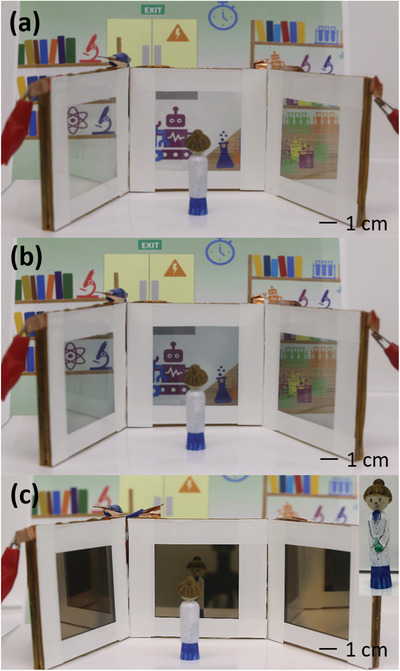
Demonstration of the three quasi‐solid‐state CuSn‐based REM devices (each panel is 64 cm^2^) programmed simultaneously at the a) transparent state (no potential), b) greyish‐blue state at −0.9 V, and c) mirror state at −1.5 V (inset: front view of Marie Curie kokeshi doll).

For the purpose of demonstration of the smart windows, three quasi‐solid‐state CuSn‐based REM devices (size of each panel is 64 cm^2^) were powered together simultaneously. **Figure** **5**a showed the setup of the three REM devices arranged side‐by‐side with the Marie Curie kokeshi doll placed in front of the device to demonstrate the mirror state and a cartoon sketch of the laboratory backdrop to show the effect of the color state. The REM devices displayed tinted state as can be seen in Figure 5b. At the mirror state (Figure 5c), transmission of light is no longer possible and hence, the device could act as privacy glass and information displays as well. Marie Curie kokeshi doll was well‐reflected on the REM device at the mirror state, as can be seen from Figure 5c (Inset: front view of Marie Curie kokeshi doll). Figure S20, Supporting Information, showed reflections of Marie Curie on all the three REM devices simultaneously.

**Figure 6:**
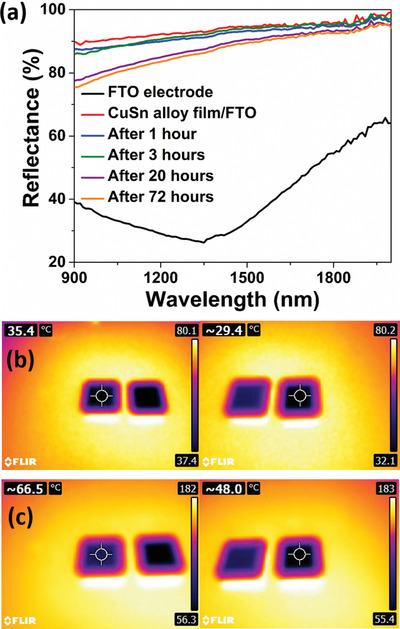
a) Reflectance spectra of the as‐deposited CuSn alloy film/FTO electrode acquired over the duration of 3 days to monitor the surface oxidation of the film in the ambient environment. Side‐by‐side comparison of thermal images of bare FTO electrode (left) and CuSn alloy film/FTO electrode heated on a hotplate at b) 80 °C and c) 180 °C.

Another noteworthy property of the CuSn alloy film is the heat blocking ability. From the reflectance spectra in **Figure** **6**a, the CuSn alloy film showed reflectance modulation of 54.9% (taken at the wavelength of 1000 nm), and increased to 61.8% at 1500 nm. Figure 6b shows the side‐by‐side thermal images of the bare FTO on glass substrate (left) and CuSn alloy film electrodeposited on the FTO on glass substrate captured by the IR camera, when heated at 80 °C on the hotplate for 5 min. The color comparison illustrates that the CuSn alloy film blocks NIR heat effectively, as it showed a colder surface temperature of 29.4 °C with 6 °C temperature difference compared to the bare FTO electrode (35.4 °C). At the elevated temperature of 180 °C, CuSn alloy film showed a larger temperature difference of 18.5 °C compared to the bare FTO electrode, as can be seen from Figure 6c. This marks the significant NIR blocking property of the CuSn alloy film, which is highly attractive for applications, such as, smart windows for building fenestration, camouflage, and thermal control. The real time temperature‐control experiments are provided in Figure S21, Supporting Information. We also studied the CuSn film stability in ambient environment for 3 days, in which the film no longer suffered severe surface oxidation (Figure 6a). At the NIR range, the role of Sn in reducing the surface oxidation of Cu film is more evident as the reflectivity of CuSn film only dropped by 2.9% at 2000 nm and 4.5% at 1500 nm after being left in the ambient environment for 3 days. On a different note, the electrodeposited CuSn alloy film had better protection against surface oxidation in the ambient environment compared to Cu film. In Figure S22, Supporting Information, the CuSn film showed minimal surface oxidation with a 0.7% drop in the film reflectivity compared to Cu film, which showed a 6.8% drop in reflectivity at 1500 nm after 3 h. Overall, the quasi‐solid‐state CuSn‐based REM device showed excellent cycling stability in both transmittance (2400 cycles) and reflectance (1000 cycles) modes compared with other literatures as shown in Table S2, Supporting Information. In addition, the memory effect of the device is also competitive, retaining the mirror state for 100 min with application of potential for only 3 min.

## 3 Conclusion

In this study, we describe a quasi‐solid‐state CuSn‐based REM device, which works based on the mechanism of Cu and Sn electrodeposition and dissolution. Via the alloying approach, the REM device demonstrated faster coloration speed, reduction in surface oxidation of the film, improvement in the reflectivity of film, and enhanced device cycling stability. The REM device exhibited cycling stability of 2400 cycles in the transmittance mode and achieved 1000 cycles in the reflectance mode without significant degradation. In other word, the REM device demonstrated tri‐optical (transparent, colored, and mirror) states in a single device. Additionally, the CuSn alloy film possessed good NIR blocking property with 18.5 °C temperature difference at elevated temperature of 180 °C compared to the bare substrate. An interesting aspect is that the employed CuSn is comparably cheap and less toxic than other materials.^[^ ^26^ ^]^ The robust REM devices are expected to revolutionize electrochromic applications to address diverse needs, such as thermal‐controlled fenestrations, industrial cooling, electronic displays, advertising, and camouflage.

## 4 Experimental Section

##### Preparation of CuSn Electrolyte and Quasi‐Solid‐State CuSn Electrolyte

Copper (II) chloride, tin (II) chloride, dimethyl sulfoxide (DMSO), lithium bis(trifluoromethanesulfonyl)imide (LiTFSI), potassium iodide, and PVA were used as purchased without further purification. For the preparation of CuSn electrolyte, CuCl_2_ (80 mm), SnCl_2_ (20 mm), LiTFSI (250 mm), and KI (0.6 mm) were dissolved in sequence in DMSO by magnetic stirring. The quasi‐solid‐state CuSn electrolyte can be prepared by adding PVA (2.5 wt%, *M*_w_ = 146000–186000) as the polymer host and stirred at 120 °C until a homogeneous quasi‐solid‐state electrolyte is obtained. FTO electrodes (15 Ω/□) were used as the transparent conducting electrodes in all the electrochemical studies as well as device fabrication.

##### Fabrication of REM Device

The REM device was fabricated using FTOs as both the working and counter electrodes with Ag wire as the reference electrode. The quasi‐solid‐state CuSn electrolyte was injected between the two FTO electrodes. For the electrochromic behavior analysis, the device active area was 4.0 × 1.5 cm^2^. For the purpose of demonstration, three large‐scale REM devices of 8.0 × 8.0 cm^2^ were fabricated and tested simultaneously. 3D‐printed Marie Curie kokeshi doll was used to demonstrate the mirror state of the quasi‐solid‐state CuSn‐based REM devices with the cartoon sketch of the laboratory backdrop to show the color state effect.

##### Material Characterizations

The XRD (Grazing Incidence XRD (GIXRD), Shimadzu discover diffractometer with Cu Kα‐radiation (λ = 1.5406 Å) and field emission scanning electron microscopy (FESEM, Model Supra 55, Carl Zeiss) were employed to characterize the crystal structure and morphology of the CuSn alloy film. A Kratos Analytical Axis UltraDLD UHV spectrometer with a monochromatized Al Kα X‐ray source (1486.6 eV) was used for XPS measurements. Thermal images were captured using the IR camera (FLIR E4, spectral range: 7.5–13 µm). The dynamic rheological measurements were performed with Anton Paar Physica CMR 501 rheometer using a 10 mm parallel plate geometry and measuring gap size of 1 mm. Constant amplitudes of deformation, 1% strain, were prepared during each frequency sweep of 0.1–10 Hz. The frequency‐sweep measurements were performed within the linear range, to obtain the storage modulus (G′) and loss modulus (G″) of the quasi‐solid‐state CuSn electrolyte.

##### Electrochemical Characterizations

The electrochemical tests were conducted using a three‐electrode setup in the CuSn electrolyte using Autolab PGSTAT30 potentiostat. Pt electrode and Ag/AgCl electrode were used as the counter electrode and reference electrode. The EIS measurement was conducted by applying AC voltage with 10 mV amplitude in a frequency range of 0.1–100 kHz at open circuit voltage. The in situ analyses were performed using UV–vis–NIR spectrophotometry (Lambda 950, Perkin Elmer) and Autolab potentiostat to obtain both the transmittance and reflectance spectra as well as the kinetic spectra for both the switching test and durability test.

## Conflict of Interest

The authors declare no conflict of interest.

## Supporting information

Supporting Information
