# Supplementary material for: A Quasi‐Solid‐State Tristate Reversible Electrochemical Mirror Device with Enhanced Stability
Source: Adv Sci (Weinh). 2020 May 12;7(13):1903198. doi: 10.1002/advs.201903198 (PMC7341104; doi:10.1002/advs.201903198)
Supplement: Supplementary file 1 — Supporting Information [file ADVS-7-1903198-s001.pdf]

Copyright WILEY-VCH Verlag GmbH & Co. KGaA, 69469 Weinheim, Germany, 2020.

Supporting Information

for *Adv. Sci.,* DOI: 10.1002/advs.201903198

A Quasi-Solid-State Tristate Reversible Electrochemical Mirror Device with Enhanced Stability

*Alice Lee-Sie Eh, Jingwei Chen, Shu Hearn Yu, Gurunathan Thangavel, Xinran Zhou, Guofa Cai, Shaohui Li, Daniel H. C. Chua, and Pooi See Lee**

Copyright WILEY-VCH Verlag GmbH & Co. KGaA, 69469 Weinheim, Germany, 2020.

Supporting Information

Quasi-Solid-State Tristate Reversible Electrochemical Mirror (REM) Device with Enhanced Stability

*Alice Lee-Sie Eh, Jingwei Chen, Shu Hearn Yu, Gurunathan Thangavel, Xinran Zhou, Guofa Cai, Shaohui Li, Daniel H. C. Chua, and Pooi See Lee**

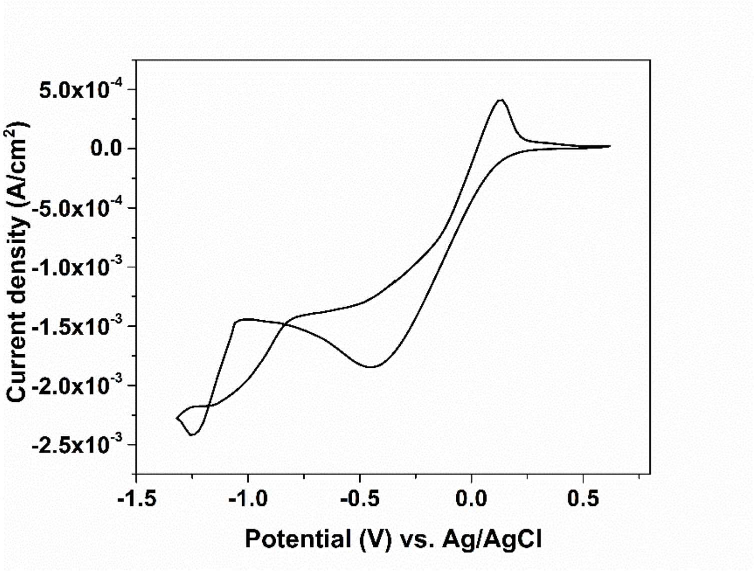

Figure S1. Cyclic voltammetry of the Cu film electrodeposition/dissolution on the FTO working electrode in the electrolyte in the potential range from – 1.3 V to + 0.6 V *vs*. Ag/AgCl.

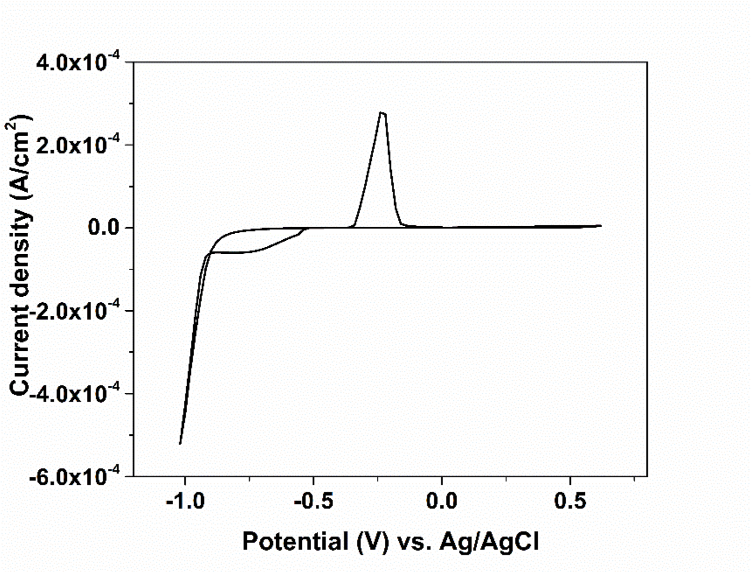

Figure S2. Cyclic voltammetry of the Sn film electrodeposition/dissolution on the FTO working electrode in the electrolyte in the potential range from – 1.0 V to + 0.6 V *vs*. Ag/AgCl.

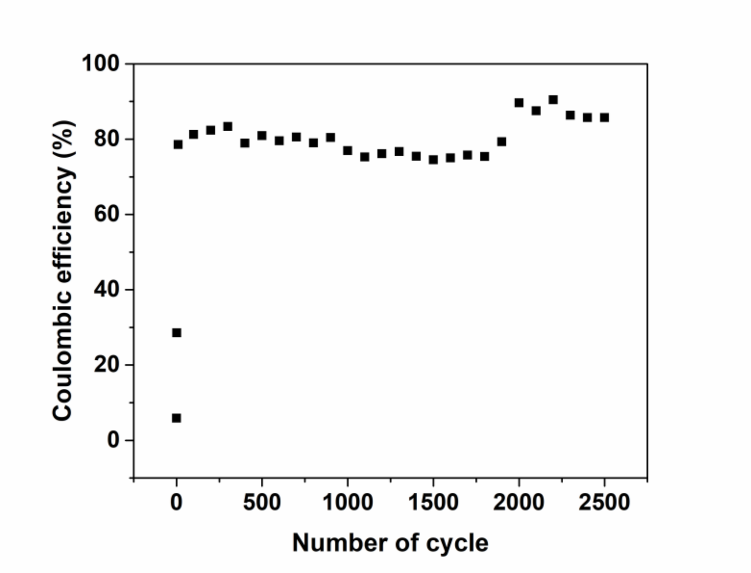

Figure S3. Coulombic efficiency of CuSn alloy film electrodeposition/dissolution in the quasi-solid-state electrolyte (calculated based on cyclic voltammogram).

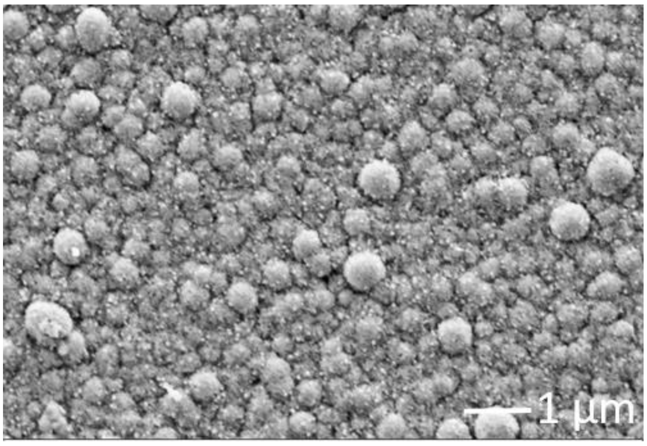

Figure S4. High-magnification FESEM image of the electrodeposited CuSn film on the FTO electrode from the CuSn electrolyte at – 1.5 V for 180 s.

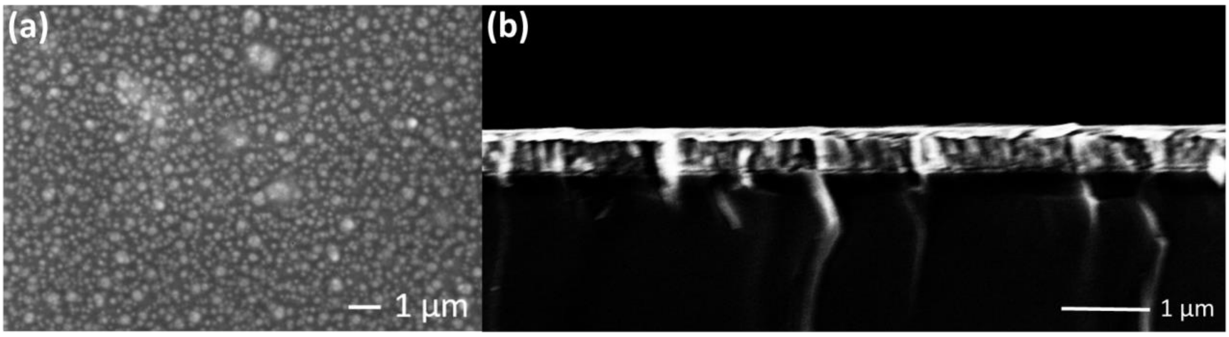

Figure S5. (a) High-magnification FESEM image of the electrodeposited CuSn film on the FTO electrode; (b) cross-section of the electrodeposited CuSn film/FTO electrode from the quasi-solid-state CuSn electrolyte at – 1.5 V for 60 s.

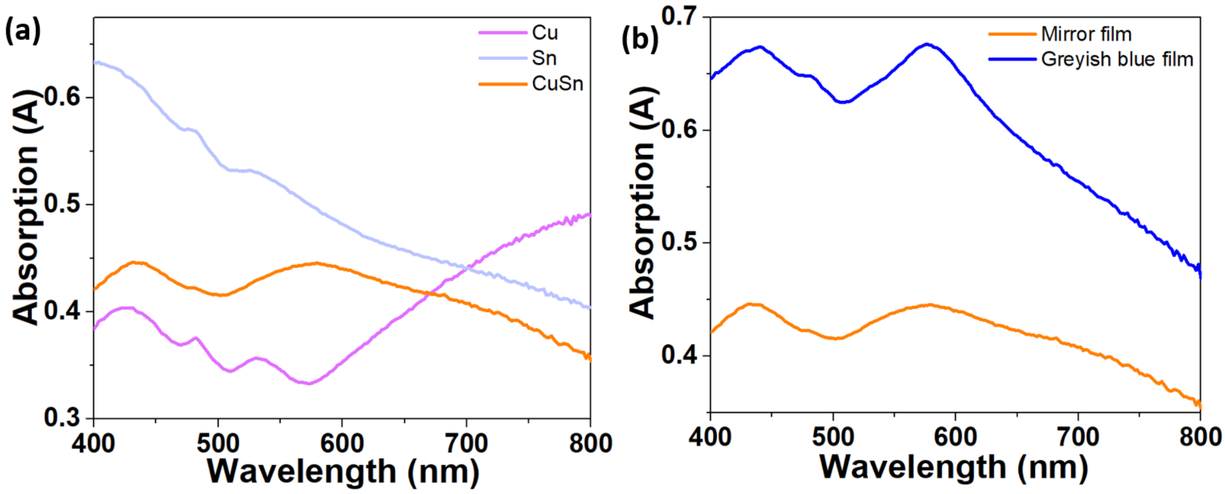

Figure S6. (a) Absorption of the mirror states of the Cu, Sn, and CuSn films; (b) absorption of the mirror film and greyish blue film from the CuSn electrolyte.

The absorption spectra of both greyish-blue and mirror films were investigated to understand the characteristic bands related to Cu transitions and Sn charge transfer. Firstly, the absorption spectra of all the mirror films (pure Cu, pure Sn, and CuSn samples) were investigated to identify the corresponding absorption peaks. From Figure S6a, all the mirror films show a common absorption peak at 481.8 nm. The pure Cu film has additional peaks at 426.2 nm and 531.1 nm whereas pure Sn film has a peak at 525.4 nm. Alloying of Sn with Cu results in charge transfer from Sn to Cu (*Nature Catalysis, 2019, 2, 55*). In CuSn film, the Cu peak has red-shifted to 435.9 nm. The CuSn sample has a characteristic absorption peak at 579.4 nm (CuSn ≈ 580-590 nm in *Journal of Electronic Materials, 2015, 44, 1175*), which is not observed in both Cu and Sn samples, further confirming the formation of CuSn alloy. The broad absorption feature observed for the CuSn after 600 nm is due to the presence of Cu^{2+} ions which absorb broadly owing to $^2E \rightarrow {}^2T_2$ intra-configuration (d-d) transitions (*Spectrochimica Acta Part A: Molecular and Biomolecular Spectroscopy, 2020, 226, 117546; Journal of Electronic Materials, 2015, 44, 1175*). For CuSn samples, both the mirror film and greyish-blue film show similar absorption spectra, but with peak shifting (Figure S6b). For the greyish-blue film, the Cu peak (Cu^+ corresponding to Cu_2O and $Cu(ClO_4)_2$ is located (red-

shifted) at 439.9 nm (Cu_2O absorption spectra: 434 nm as reported in *Nanoscale Research Letters, 2012, 7, 347*). The presence of Cu^+ is revealed by the red shift, in connection with $3d^{10} \rightarrow 3d^9 4s^1$ electronic transitions, which although parity-forbidden in nature, become partially allowed in solid film due to ion-lattice interactions (*Spectrochimica Acta Part A: Molecular and Biomolecular Spectroscopy, 2020, 226, 117546; Journal of Electronic Materials, 2015, 44, 1175).*

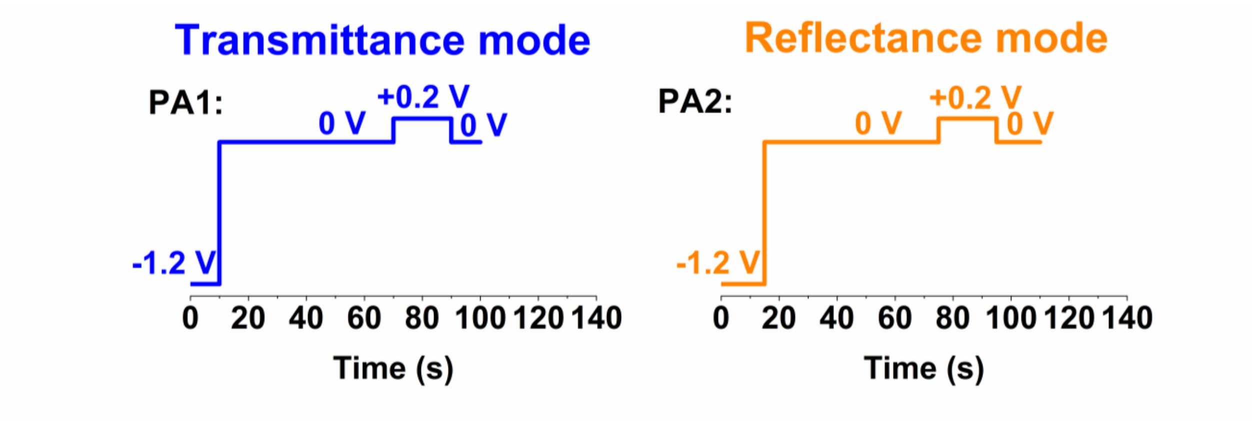

Figure S7. The potential algorithms (PA1 and PA2) in waveform used for the cycling performance of the quasi-solid-state CuSn-based REM device in the (a) transmittance mode and (b) reflectance mode.

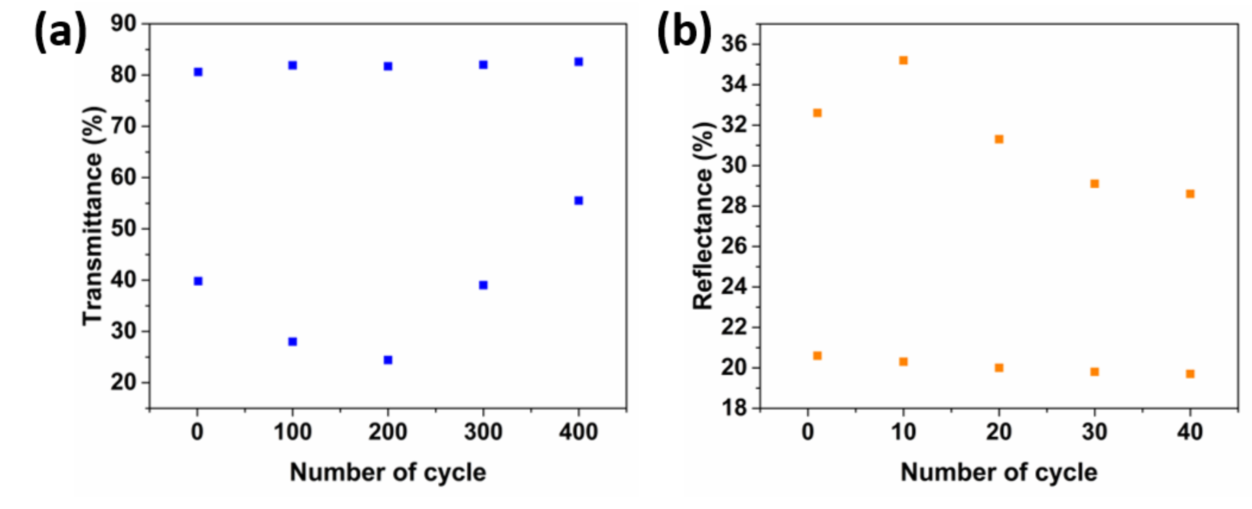

Figure S8. (a) Cycling performance of the pure Cu-based REM device for 400 cycles using PA1 at 550 nm in the transmittance mode; (b) cycling performance of the pure Cu-based REM device for 40 cycles using PA2 at 660 nm in the reflectance mode.

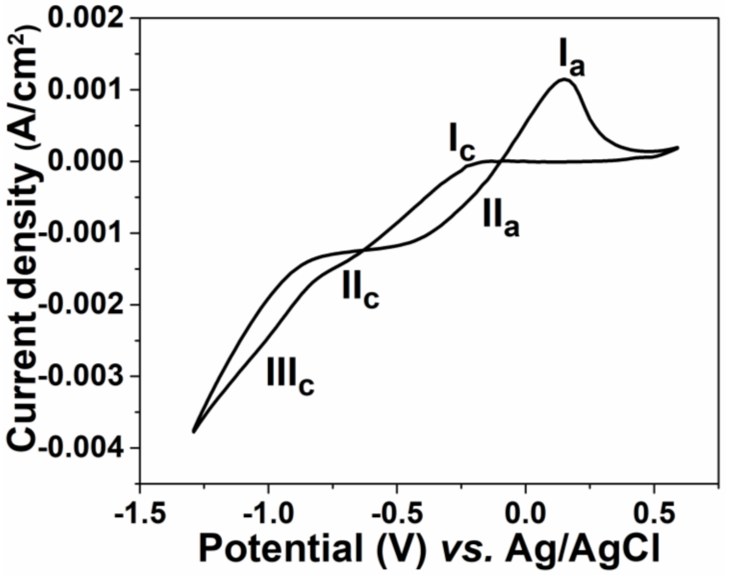

Figure S9. Cyclic voltammogram of the CuSn alloy film electrodeposition/dissolution on the FTO working electrode after the long-time cycling (2,400 cycles) in the electrolyte in the potential range from -1.30 V to +0.60 V *vs.* Ag/AgCl.

To investigate the electrochemical reduction and oxidation behaviors of the CuSn film after the long-time cycling, CV analysis of the CuSn electrodeposition/dissolution on the FTO electrode was performed (Figure S9). CV was conducted at a scan rate of 20 mV s^{-1} from -1.30 to +0.60 V (*vs.* Ag/AgCl) in a three-electrode configuration with platinum electrode as the counter electrode. The respective CVs of the CuSn before and after 2,400 cycles were compared. From Figure 4c in the main text, there is a slight drop in transmittance at the bleached state at 2,400th cycle. The device experienced difficulty in achieving complete film dissolution. The presence of undissolved nanoparticles acted as the nucleation layer for the subsequent electrodeposition of Cu and Sn. Compared to the initial CV before device cycling (Figure 1a in the text), the first cathodic peak in Figure S9, $I_c \approx -0.23$ V shifted to less negative potential, which facilitates the electrochemical reduction of Cu^{2+} ions to Cu^{+} ions. Nonetheless, the second cathodic peak, $II_c \approx -0.71$ V and third cathodic peak $III_c \approx -1.01$ V shifted towards more negative potential, indicating the increased challenge to achieve electrochemical reduction of Sn^{+} to Sn^{0} and Cu^{+} to Cu^{0}. This can be explained by the higher transmittance of the CuSn film as can been seen in Figure 4c in the text. The two cathodic crossovers were observed, showing a typical nucleation overpotential (*AIP Conference Proceedings, 2013, 1571, 120; Analytical Chemistry Insights, 2016, 11, 1-11*). The presence of the crossover in the CV is typical of the formation of a new phase involving a nucleation process and growth (*Analytical Chemistry Insights, 2016, 11, 1-11*). Compared to the initial

CV, both the anodic peaks, $II_a \approx -0.16$ V and $I_a \approx 0.15$ V shifted towards a more positive potential. Hence, the aged device required a higher oxidation potential in order to achieve a complete film dissolution (Cu^+ to Cu^{2+}). After the long durability test of 2,400 cycles, the aged REM device experienced degradation in the electrochromic performance as confirmed by the CV analysis. In short, the aged device required a more negative reduction potential to achieve the mirror state and a more positive oxidation potential to achieve complete film dissolution, which explained the drop in the transmittance modulation at the 2,400th cycle.

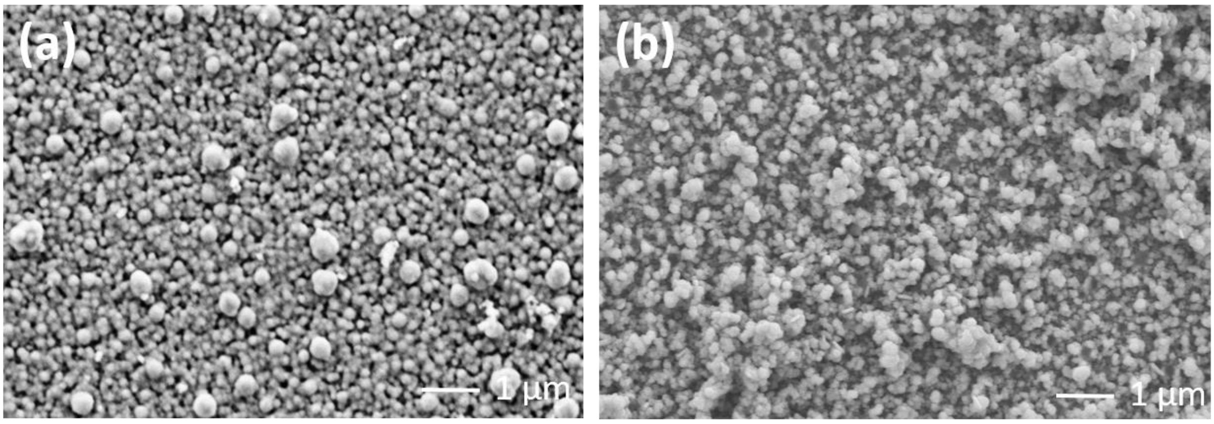

Figure S10. High-magnification FESEM images of the electrodeposited CuSn film on the FTO electrode at -1.5 V for 60 s (a) before cycling (b) after cycling (2,400 cycles).

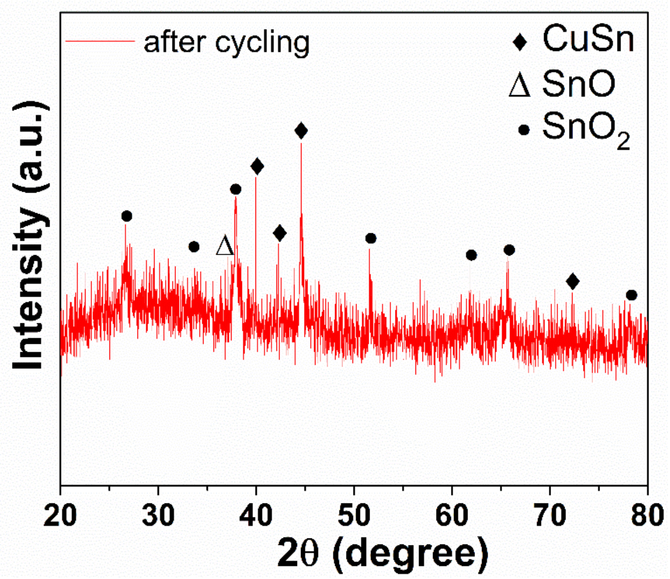

Figure S11. XRD pattern of the electrodeposited CuSn alloy mirror film/FTO electrode after cycling (2,400 cycles).

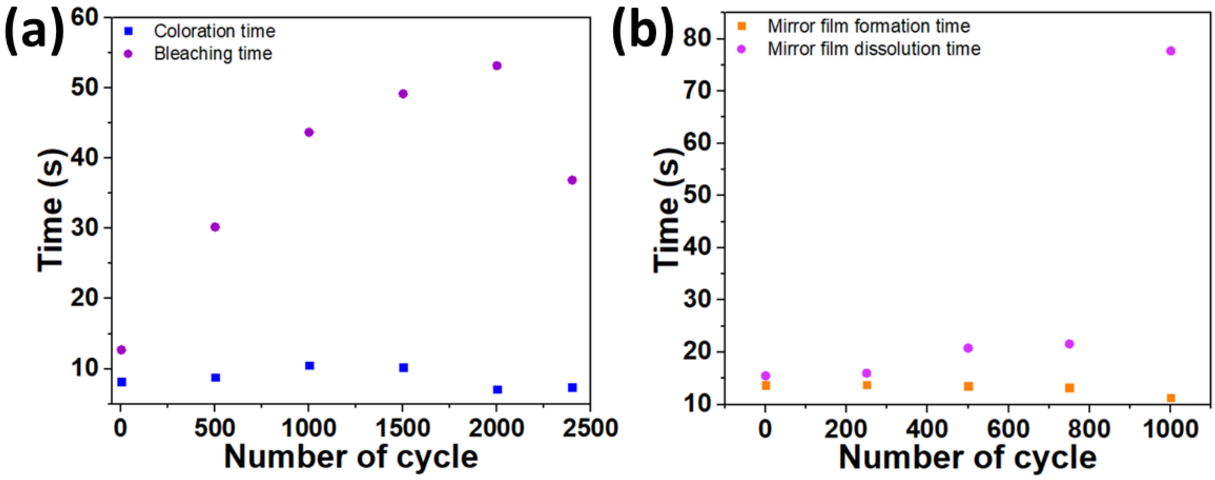

Figure S12. Cycle kinetics of the quasi-solid-state CuSn-based REM device at different switching cycles in the (a) transmittance and (b) reflectance modes.

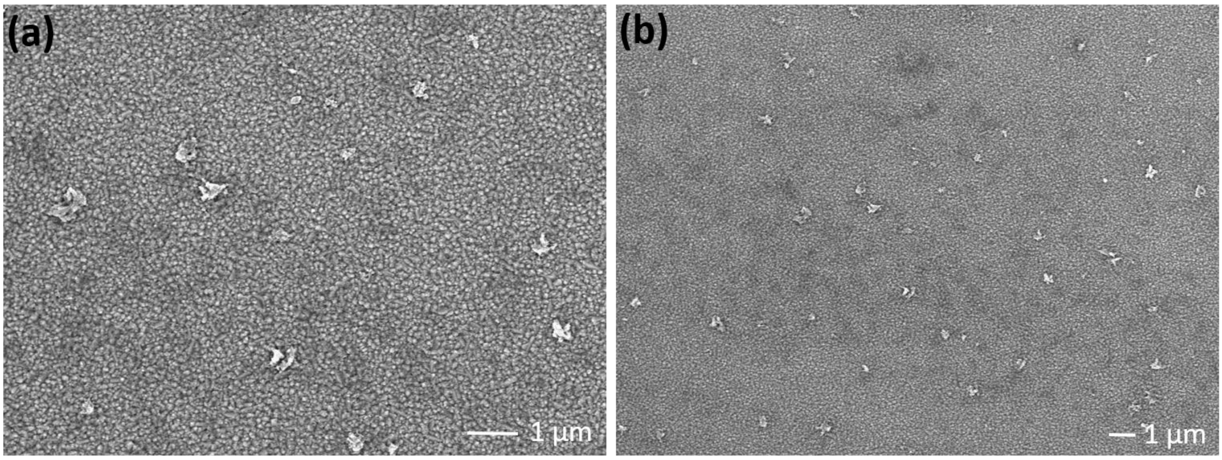

Figure S13. (a) and (b): FESEM images of the film after the dissolution step (after 20 cycles) at different magnifications, showing presence of nucleation layer on the FTO electrode.

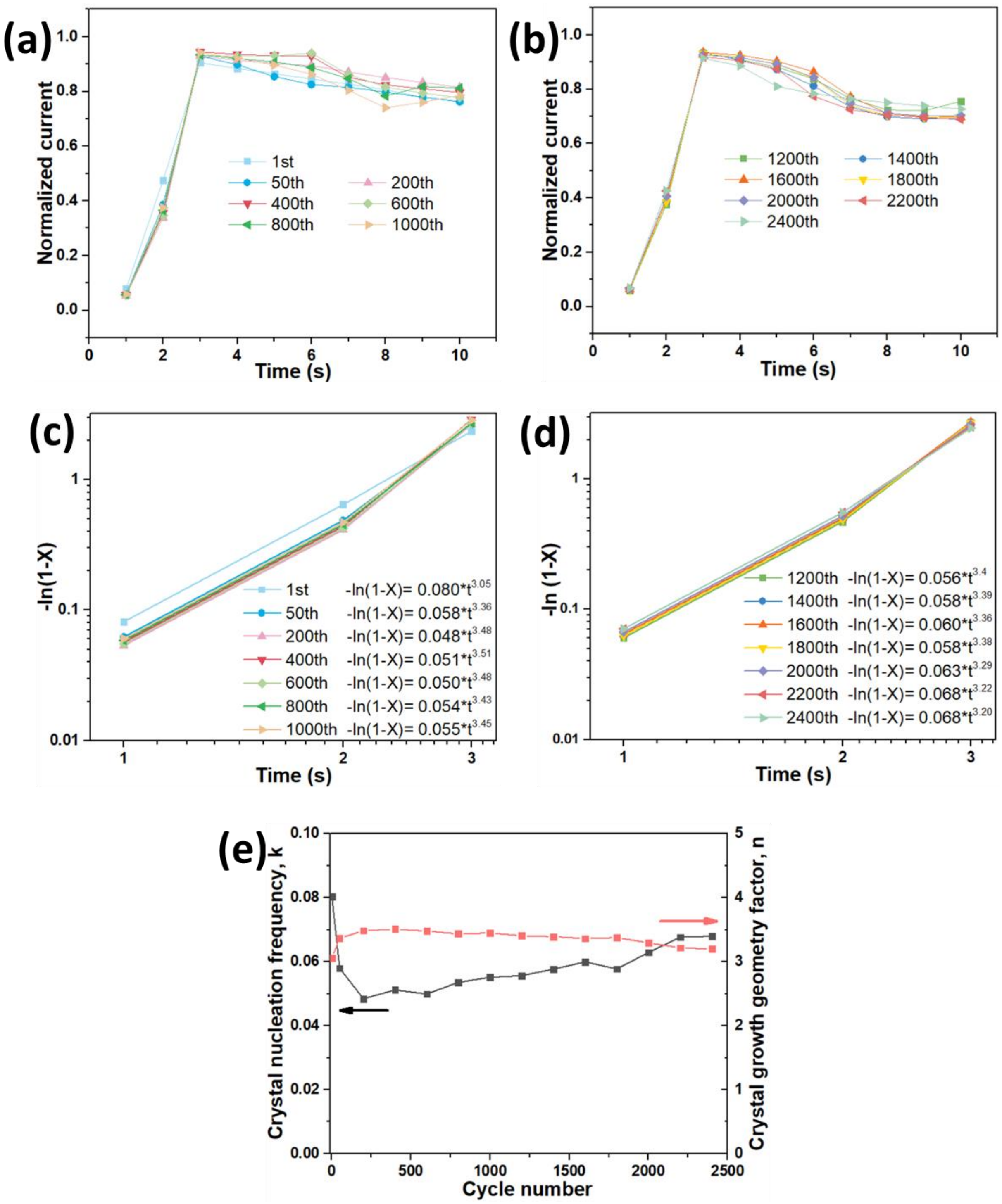

Figure S14. Normalized current-time (s) curves for electrodeposition at different cycle numbers. (a) 1st to 1000th cycles, (b) 1200th to 2400th cycles. Double logarithmic plot of $-\ln(1-X)$ *vs*. t derived from the current increase process (stage 1) at different cycle numbers. (c) 1st to 1000th cycles, (d) 1200th to 2400th cycles. X is the current normalized over the saturation current. (e) Extracted values of n (crystal growth geometry factor) and k (crystal nucleation frequency) during the cycling process.

During the cycling process, -1.2 V for 10 s (fixed time) was applied for the film formation process in every cycle. We have extracted the i-t (current-time) curves (the absolute current values) at different cycles for the total of 2,400 cycling process as shown in Figure S14a-b. It can be seen that there is a sudden increase of the current (stage 1, first three seconds) for all the cycles. This is followed by a current saturation stage (stage 2, subsequent seconds up to 10 sec) for all 2400 cycles. In order to evaluate the nucleation frequency and Avrami coefficient values during the cycling process, Johnson–Mehl–Avrami–Kolmogorov (JMAK) analysis, $X = 1 - \exp(-kt^n)$ was applied for stage 1, X is the reaction ratio, t is the reaction time, n is the crystal growth geometry factor, and k is the crystal nucleation frequency (*Crystals, 2019, 9, 142; Journal of Physics D: Applied Physics, 2011, 44, 443001*). Current values were normalized over the saturation current and the double logarithmic plots of -ln(1-X) *vs* time for different cycles are presented as shown in Figure S14c-d. The values of parameters n (crystal growth geometry factor) and k (crystal nucleation frequency) were estimated from the slope and the intercept and has been consolidated into Figure S14e. This is following the analysis of $\ln[-\ln(1 - X)] = \ln k + n \ln t$. It can be seen that the n values of different cycles are all slightly higher than 3, indicating that the initial film formation is due to the unhindered 3-dimensional (3D) growth of nuclei (*Crystals, 2019, 9, 142)*. Subsequently, the n value slightly reduces, which is probably due to the accumulation of nuclei.

An initial dip in the crystal nucleation frequency (k) in the first 200 cycles likely related to the non-dissolved nanoparticles resulting in reduced number of new nucleation sites (Figure S14e). The gradual increase relates to the more evenly distributed nucleation sites (undissolved particles) accumulated after the first 200 cycles, leading to lower transmittance of the films deposited, therefore higher optical modulation in Figure 4c (main text). The crystal growth geometry factor (n) is ≈3, indicating three-dimensionally growth (3D) of nuclei. The presence of non-dissolved nanoparticles (nuclei) has been verified as shown in Figure S13a-b. The non-dissolved nuclei in one way assists the 3D growth of crystals and film

formation in the first few hundreds of cycles, yet in subsequent cycles accumulated number of nuclei impede the dissolution process and eventually leads to device degradation.

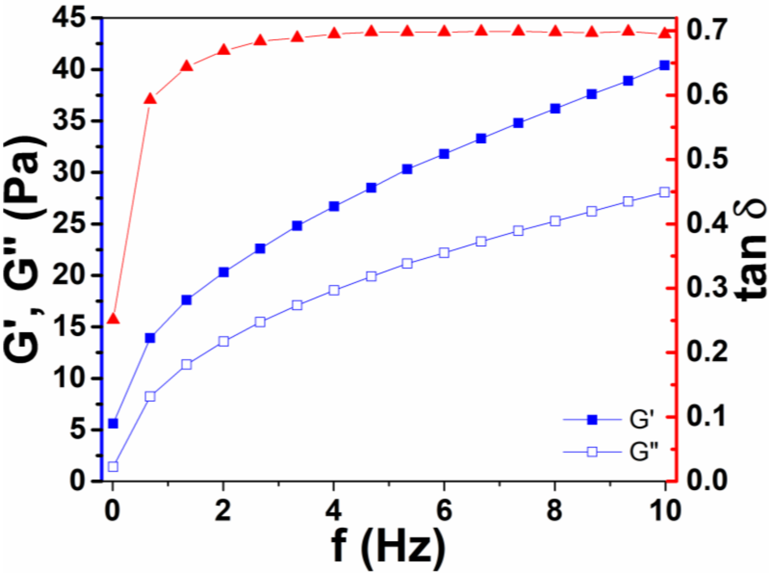

Figure S15. Rheological response (shear storage modulus, G', loss modulus, G", and tan δ with frequency) of quasi-solid-state CuSn electrolyte. The intersection of G' and G" is defined as frequency-dependent gelation as determined by rheology. The initial decreases in G'/G" can be explained by the decrease in viscosity with increasing temperature.

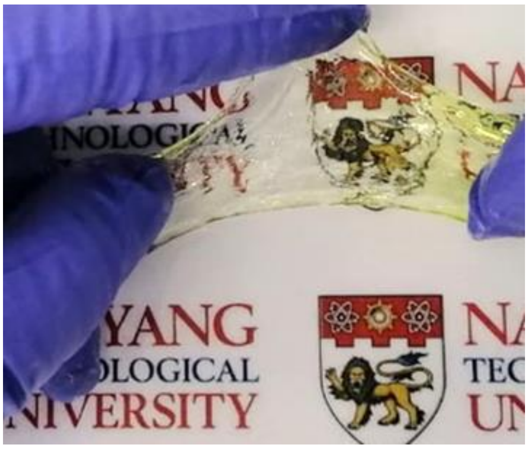

Figure S16. Slime-like behavior of the quasi-solid-state CuSn electrolyte.

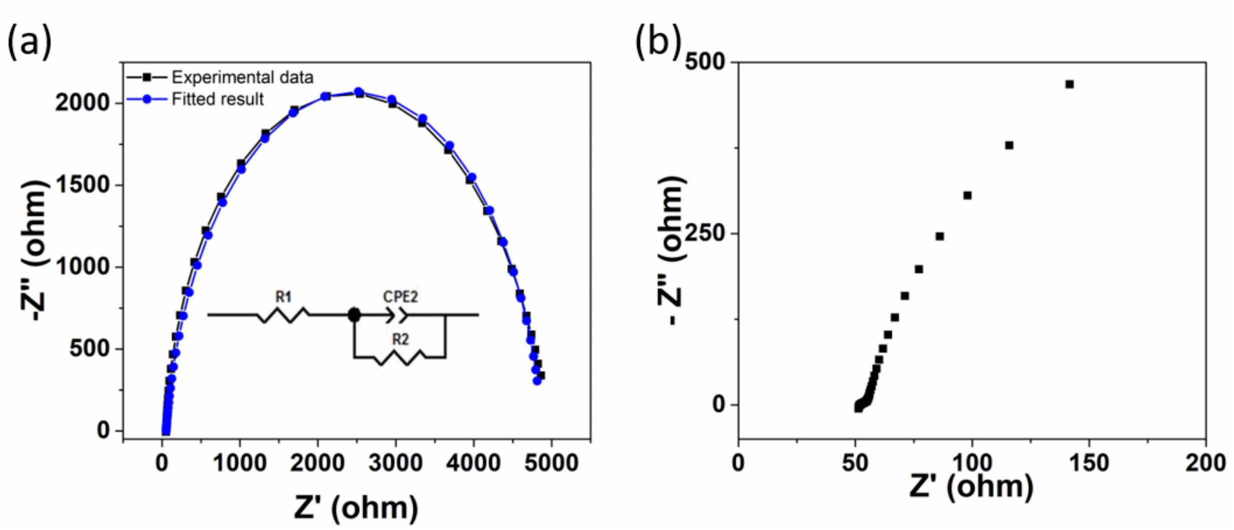

Figure S17. (a) Fitted EIS spectra obtained by the ZView software using the experimental data. Inset: the equivalent circuit used to model the EIS of the quasi-solid-state CuSn electrolyte determined by the ZView software; (b) enlarged area of the high frequency region of the Nyquist plot of the CuSn electrolyte.

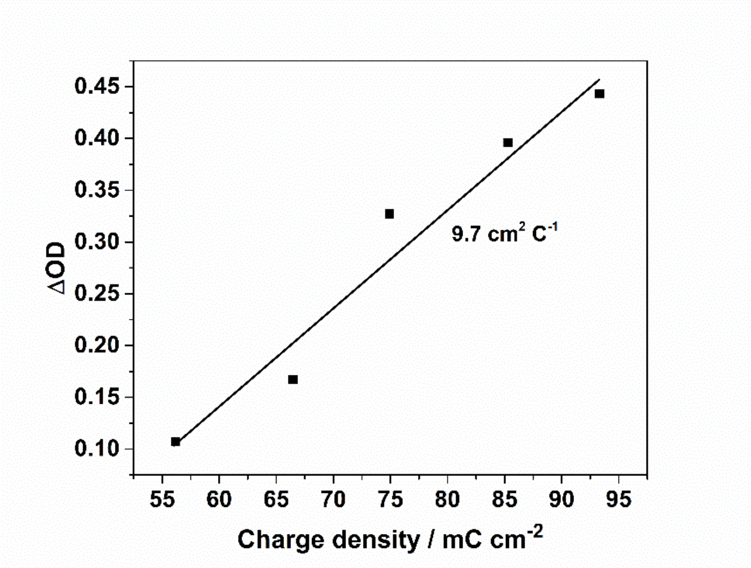

Figure S18. Reflectance efficiency of the quasi-solid-state CuSn-based REM device.

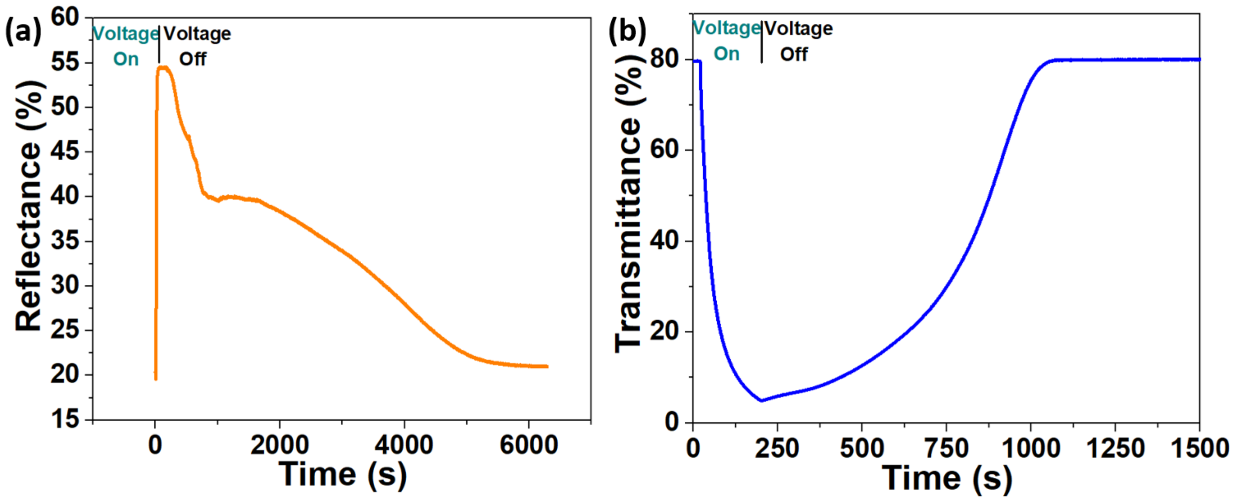

Figure S19. (a) Memory effect retention of the mirror state of the quasi-solid-state CuSn-based REM device in the reflectance mode (at 660 nm) during the voltage-off state after applying a potential of -1.5 V for 3 minutes; (b) Memory effect retention of the greyish-blue state of the quasi-solid-state CuSn-based REM device in the transmittance mode (at 550 nm) during the voltage-off state after applying a potential of -0.9 V for 3 minutes.

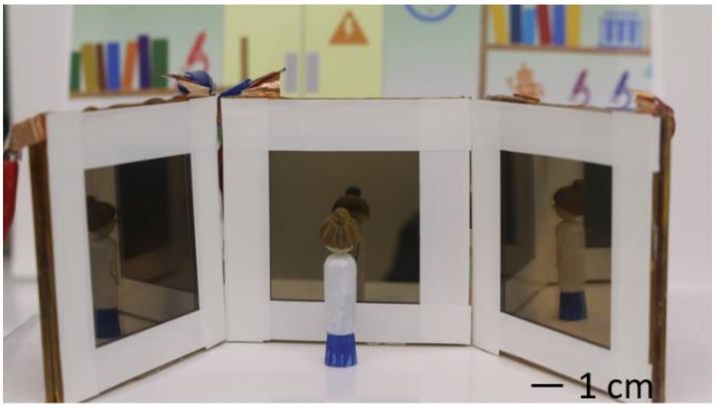

Figure S20. Reflection of Marie Curie kokeshi doll on all the three quasi-solid-state CuSn-based REM devices at the mirror state.

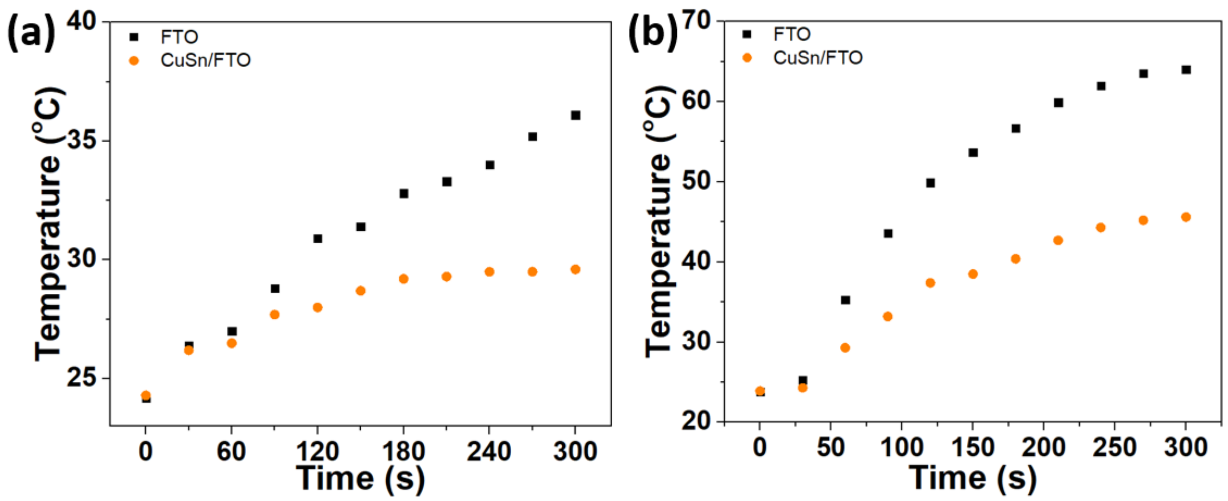

Figure S21. Real-time temperature-control experiments which showed the temperature captured by the IR camera when both bare FTO electrode and CuSn alloy film/FTO electrode were heated on a hotplate at (a) 80 °C and (b) 180 °C.

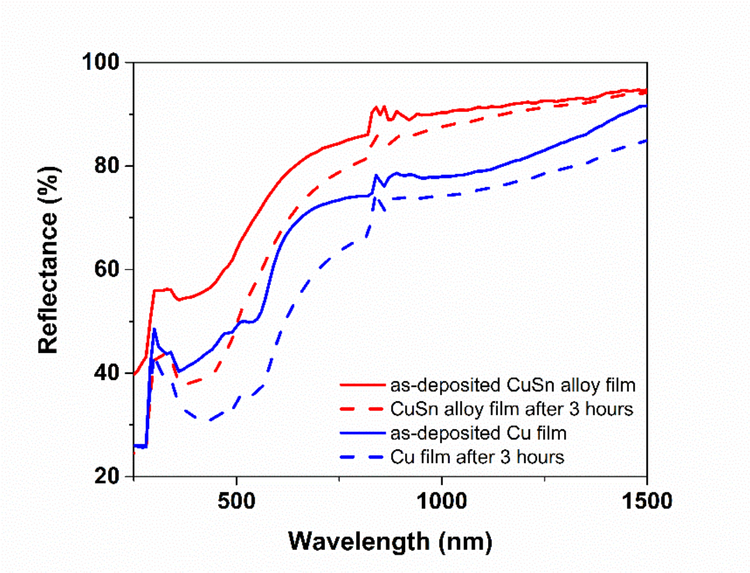

Figure S22. Change in reflectance of the as-deposited CuSn alloy film and Cu film in the ambient environment over the duration of 3 hours.

Table S1. XPS analysis of the atomic ratios of the elements found in the CuSn mirror film and greyish-blue film

	Atomic ratio (%)				
	Cu	Sn	O	Cl	C
**CuSn mirror film**	8.53	13.11	37.34	2.24	38.66
**Greyish-blue film**	6.10	4.12	50.36	1.52	37.91

Table S2. Performance comparison between reversible electrochemical mirror devices

Material	No. of state	Cycling in T mode (No. of cycles)	Cycling in R mode (No. of cycles)	Switching in T mode	Switching in R mode	Memory effect	Reference
Ag	3	2,500	N.A.	N.A.	N.A.	N.A.	[1]
Ag	2	N.A.	24*	N.A.	Mirror formation 60-180 s; mirror dissolution < 60 s^	120 min (apply -2.5 V, 30 min)	[2]
Ag	2	31*	32*	25.1 s~	97.9 s~	20 min (apply -2.5 V, 3 min)	[3]
Ag	3	1,500	N.A.	N.A.	N.A.	N.A.	[4]
Cu	3	200	N.A.	Coloration 24.2 s; bleaching 17.4 s	Mirror formation 23.3 s; mirror dissolution 13.7 s	21 min (apply -1.8 V, 10 min)	[5] Our previous work
Cu	3	2,400	1,000	Coloration 9.8 s; bleaching 18.4 s	Mirror formation 24.7 s; mirror dissolution 30.2 s	100 min (apply -1.5 V, 3 min)	Current work

Note:
*In reference 2 and 3, the number of cycles were not stated and were obtained from the respective figures in the main text.
Switching speeds are calculated based on 90% of its full modulation between the steady colored state and bleached state (in case of reflectance: between mirror film formation and dissolution) unless stated otherwise.
^The switching speed was not provided, the time for mirror formation was given in the figure and mirror dissolution was mentioned in the text.
~Time given referred to 90 % transmittance/reflectance change, specifications not given.

Supplemental References

[1] Araki, S.; Nakamura, K.; Kobayashi K.; Tsuboi, A.; Kobayashi, N. Electrochemical optical-modulation device with reversible transformation between transparent, mirror, and black.*Advanced Materials* **2012**, 24, OP122-126, OP121.

[2] Park, C.; Seo, S.; Shin, H.; Sarwade, B. D.; Na, J.; Kim E. Switchable silver mirrors with long memory effects. *Chemical Science* **2015**, 6, pp. 596-602.

[3] Park, C.; Na, J.; Han, M.; Kim, E. Transparent Electrochemical Gratings from a Patterned Bistable Silver Mirror," *ACS Nano* **2017**, 11, pp. 6977-6984.

[4] Ye, T.; Xiang, Y.; Ji, H.; Hu, C.; Wu, G. Electrodeposition-based electrochromic devices with reversible three-state optical transformation by using titanium dioxide nanoparticle modified FTO electrode. *RSC Advances* **2016**, 30769-30775.

[5] Eh, A. L.-S.; Lin, M.-F.; Cui, M.; Cai, G.; Lee, P.S. A copper-based reversible electrochemical mirror device with switchability between transparent, blue, and mirror states. *Journal of Materials Chemistry C* **2017**, 6547-6554.
